# Palm phytoliths in subarctic Canada imply ice-free winters 48 million years ago during the late early Eocene

**DOI:** 10.1093/aob/mcaf021

**Published:** 2025-02-10

**Authors:** Peter A Siver, Alberto V Reyes, Andrzej Pisera, Serhiy D Buryak, Alexander P Wolfe

**Affiliations:** Botany Department, Connecticut College, New London, CT, 06415, USA; Department of Earth and Atmospheric Sciences, University of Alberta, Edmonton, Alberta, T6G 2E3, Canada; Institute of Paleobiology, Polish Academy of Sciences, 00–818 Warszawa, Poland; Department of Earth and Atmospheric Sciences, University of Alberta, Edmonton, Alberta, T6G 2E3, Canada; Department of Physical Sciences, MacEwan University, Edmonton, Alberta, T5J 4S2, Canada; Department of Earth and Atmospheric Sciences, University of Alberta, Edmonton, Alberta, T6G 2E3, Canada

**Keywords:** Subarctic, Eocene, Giraffe pipe, kimberlite maar, palms, phytoliths, stegmata

## Abstract

**Background and Aims:**

Phytoliths are microscopic siliceous structures produced in specific tissues by many plant families. The morphological features of phytoliths are diagnostic for many plant taxa and, given their inorganic composition, often become part of the fossil record. We used phytolith remains from lacustrine sediments to document the conclusive presence of Arecaceae (palms) in subarctic Canada during the late early Eocene (48 Ma).

**Methods:**

Palm phytoliths and aquatic microfossils were extracted from lacustrine mudstones in a drill core taken from the Giraffe kimberlite pipe locality using a combination of acid and oxidation treatments under low heat. Light microscopy and scanning electron microscopy were used to identify, examine and image the microfossils.

**Key Results:**

Spherical echinate-shaped palm phytoliths with cone-shaped surface tubercles, likely belonging to the tribe Trachycarpeae (subfamily Coryphoideae), were uncovered in 45 strata over a 37-m section of core. We further document *in situ* linear arrays of phytoliths, or stegmata, from partially decomposed palm foliage. Additionally, four aquatic organisms, largely restricted to warm subtropical and tropical localities today, were also uncovered in the same strata harbouring the palm phytoliths.

**Conclusions:**

The presence of palm phytoliths allows inference of a warm regional climate during the late early Eocene, with mean cold-month temperatures above freezing despite prolonged winter darkness. This conclusion is supported by the presence of multiple warm-water aquatic organisms that grew extensively in the maar lake. Our findings will help to document the extent and timing of perennial ice formation in the northern hemisphere during the Cenozoic. Finally, the discovery of stegmata documents that this morphological trait had evolved by early Eocene.

## INTRODUCTION

Palms are monocotyledonous flowering plants in the family Arecaceae, distributed primarily in tropical and subtropical regions around the world (Tomlinson, 2006; Eiserhardt *et al*., 2011; Muscarella *et al*., 2018). It is a large family, with especially high species diversities found in Central and South America, and in southeast Asia. In general, because palms thrive under warm and wet conditions, the vast majority of species are found in tropical rainforests. Significantly fewer species are found in both southern Europe and southern regions of the USA, and the family is lacking altogether in more northern latitudes (Eiserhardt *et al*., 2011). In the subtropical south-eastern USA, palms are largely restricted to coastal regions in states along the Gulf of Mexico to Florida, along the Atlantic coast extending north to North Carolina, and a few extending inland to approximately Tennessee (Butler and Larson, 2020).

Even though the vast majority of palms are found in climates marked by both high mean annual temperature (MAT) and high mean annual precipitation (MAP), a few species can be found under cooler and drier conditions (Reichgelt *et al*., 2018; Brightly *et al*., 2024). For example, some members of the tribe Trachycarpeae are better adapted to drier conditions, others to regions with seasonal rainfall, and a few are cool-tolerant compared with most palms (Reichgelt *et al*., 2018; Brightly *et al*., 2024). Regardless of how cool-tolerant some species may be, palms cannot withstand areas with perennial ice or sustained winter conditions below freezing. Palms, including long-lived palm trees, are unique in that their tissues are developed solely through primary growth, meaning that they lack the secondary growth typical of woody plants (Tomlinson, 2006). Because palms remain physiologically active throughout the year, do not undergo dormancy and have a high water content, they are incapable of occupying areas that experience prolonged freezing conditions or severe frost events (Greenwood and Wing, 1995; Sluijs *et al*., 2009; Archibald *et al*., 2014). In turn, this strong environmental preference has been exploited for reconstructing substantially warmer-than-present past climates in regions that are presently characterized by mean annual temperatures <10 °C and coldest month mean temperatures (CMMTs) around −10 °C, e.g. north-east Russia (Akhmetiev, 2015), south-central Alaska (e.g. Wolfe, 1977; Sunderlin *et al*., 2014), central Alberta, Canada (Greenwood and West, 2017), and Wyoming, Utah and Colorado in the interior continental USA (e.g. Knowlton, 1930; Allen, 2015)

Many members of the plant kingdom produce siliceous microstructures known as phytoliths, or plant stones (Huisman *et al*., 2018). Phytoliths are produced from soluble silica taken up by the roots and deposited either within specific cells or in extracellular locations (Piperno, 2006), and can be found in different organs depending on the plant species. Phytoliths most likely serve to strengthen the specific organ in which they are formed, and to deter herbivores and possibly pathogenic fungi (Piperno, 2006). Although the shapes of phytoliths are highly variable across the plant kingdom, they are diagnostic for specific groups of plants (e.g. Arecaceae). Because the siliceous phytoliths most often remain in soils or aquatic sediments after the plant dies and decomposes, they are routinely incorporated into the fossil record (e.g. Strömberg and McInerney, 2011).

Palms often produce large numbers of phytoliths, especially within foliar tissues, that can be diagnostic at the tribe or even genus level. Palm foliage often forms contiguous files of cells, each of which produces a phytolith. These cellular arrangements are known as stegmata and are important components of palm sclerenchyma (Marcote-Ríos *et al*., 2016). Typically, if the leaf is fully decomposed upon death, the phytoliths would remain as individual structures in the sediments or soil. However, because they are taxonomically diagnostic and become part of the fossil record, their remains are valuable indicators for reconstructing past community structure and palaeo-ecological conditions (Strömberg and McInerney, 2011; Liu *et al*., 2023). Perhaps more importantly, the presence of palm phytoliths in a fossil deposit can be effectively used to infer mean winter temperatures above freezing.

The purpose of this communication is to report the presence of palm phytoliths over an extensive period of time at a late early Eocene locality situated near the Arctic Circle in Canada. Numerous individual phytoliths, as well as intact linear files of stegmata, are documented. The phytolith remains, along with those of other warm-water organisms, are collectively used to support a warm ‘greenhouse’ climate, with mean winter air temperatures above freezing, ~48 Ma in a continental region of the Canadian subarctic.

## SITE DESCRIPTION

**Fig. 1.**
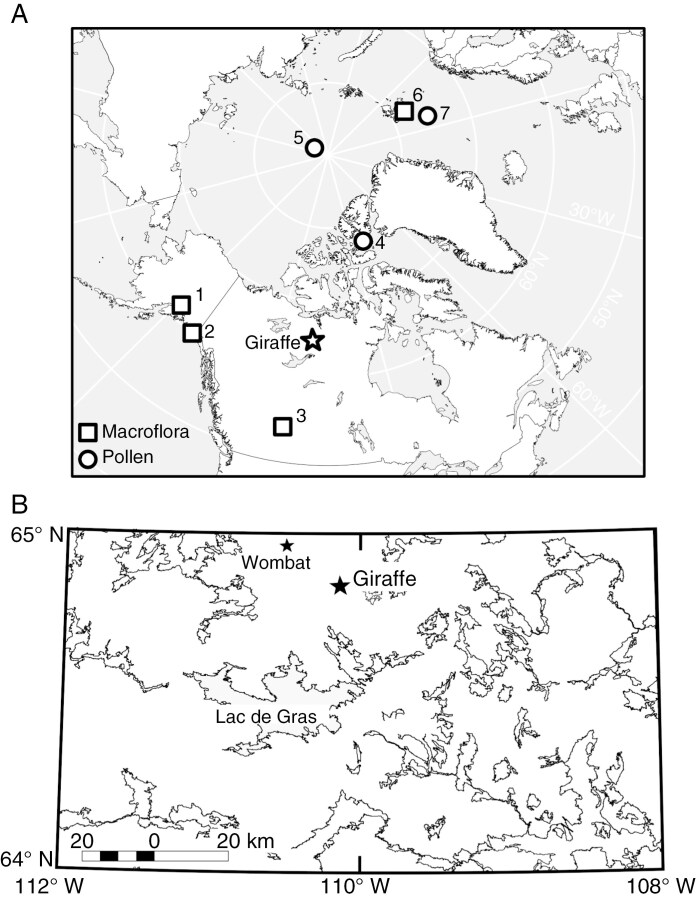
(A) Map of the circumpolar north in polar stereographic projection, indicating known Palaeogene palm localities (squares, macroflora; circles, pollen) and the Giraffe locality (star) in the Lac de Gras region, Northwest Territories, Canada. Known localities are numbered: 1, Talkeetna Mountains, Alaska (Sunderlin *et al*., 2014); 2, coastal Gulf of Alaska (Wolfe, 1977); 3, Genesee, Alberta (Greenwood and West, 2017); 4, Stenkul Fiord, Ellesmere Island (Harrington *et al*., 2012); 5, IODP Site 302-4A, Arctic Ocean (Sluijs *et al*., 2009); 6, Spitsbergen (Schweitzer, 1980); 7, ODP Site 913, Norwegian–Greenland Sea (Eldrett *et al*., 2009). Not shown are IODP Site U1356 off Wilkes Land, Antarctica (pollen; Pross *et al*., 2012) and sites in the Rocky Mountains, USA (macroflora; Allen, 2015). (B) Map of the Lac de Gras region, Northwest Territories, Canada (adapted from Stasiuk *et al.*, 2006) indicating the Giraffe and Wombat (Buryak *et al*., 2024) kimberlite maar localities.

The Giraffe locality (64.73°N, 109.75°W) comprises the post-eruptive sediment fill of a kimberlite maar in subarctic Canada (Fig. 1). Kimberlites are ultramafic volcanic rocks emplaced during phreatomagmatic eruptions that typically lead to the formation of narrow diatremes that broaden towards the surface, termed kimberlite ‘pipes’ (e.g. Giuliani and Pearson, 2019). The kimberlite diatreme is typically infilled quasi-instantaneously by deposition of the eruptive materials and adjacent country rock. Rarely, post-eruptive craters may form following kimberlite emplacement and become host to maars, the term for lakes that form in volcanic craters. Sediment fills in maars are generally derived from marginal mass movements, allochthonous deposits from streams and creeks flowing into the crater, direct atmospheric deposition, and/or autochthonous production of organic matter, carbonates and silica within the lake (Büchel, 1993). Many kimberlite maars in subarctic Canada are typically small in diameter but relatively deep, which may promote bottom-water anoxia and outstanding preservation of fossils and other organic matter (e.g. Sabel *et al.*, 2005; Hamblin, 2015; Smith *et al.*, 2018; Siver and Lott, 2023).

**Fig. 2.**
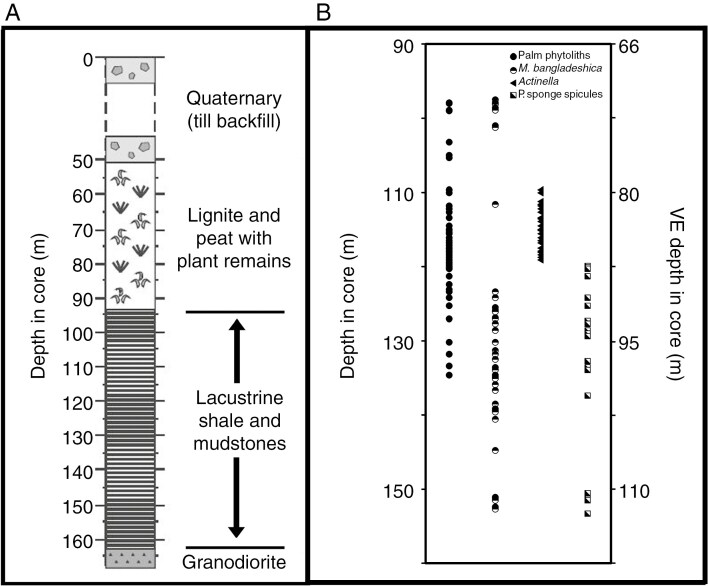
(A) Summary of the stratigraphic log of Giraffe drill core BHP99-01. (B) Distribution of palm phytoliths, *Mallomonas bangladeshica*, *Actinella* species and potamolepid sponges (identified as ‘P. sponge spicules’) throughout the lacustrine phase of the core taken at the Giraffe pipe locality. Each symbol represents the presence of fossil remains at a specific depth in the core and verified with scanning electron microscopy. Both the depth along the core (left axis) and the VE depth (right axis) with respect to the surface are given.

The Giraffe kimberlite maar is within the Lac de Gras kimberlite field, in the Slave Craton of the Canadian Shield in Northwest Territories, Canada (Fig. 1). Periods of kimberlite magmatism in the Lac de Gras region are thought to fall into five pulses centred on ~80, ~70, 60, 54 and 48 Ma (Creaser *et al*., 2004; Sarkar *et al*., 2024). Xenoliths within kimberlite diatremes indicate that the Lac de Gras region was covered by Middle Devonian and Cretaceous–Palaeogene sedimentary rocks at various times (e.g. Cookenboo *et al*., 1998; Sweet *et al*., 2003), though presently the region is almost completely devoid of Phanerozoic cover rocks. The Giraffe kimberlite locality has been intersected by three diamond exploration drill cores; sediments from core BHP99-01, which are the subject of this report, are archived at the Geological Survey of Canada (Calgary). Core BHP99-01 was drilled in 1999 at 47° relative to the vertical axis, and is 163 m long (Fig. 2A). The post-eruptive crater infill sediments in BHP99-01 comprise ~80 vertical-equivalent (VE) m of undisturbed, organic-rich sedimentary fill, with at least ~50 VE m of lacustrine sediment overlain by ~30 VE m of peat. The base of the angled drill core intersected granitic country rock, so the true thickness of the lacustrine fill is unknown (Buryak *et al*., 2024). The stratigraphy, sedimentology and organic geochemistry of the Giraffe maar sediments have been described by Stasiuk *et al*. (2006) and Hamblin (2015), and a reconstruction of the maar lake based on numerous microfossil remains was made by Siver and Lott (2023).

Creaser *et al*. (2004) used an Rb–Sr age estimate based on macrocrystal phlogopite to set the emplacement age of the Giraffe kimberlite at 47.8 ± 1.4 Ma. More recently, geochronology for the Giraffe maar lacustrine and peat sediments has been derived from distal rhyolitic tephra beds (Westgate and Bray, 2021; Buryak *et al*., 2024). The tephra layers were originally dated to 37.8 ± 4.0 Ma using glass fission-track techniques (Doria *et al*., 2011; Wolfe *et al*., 2017). However, reliable fission-track dating for volcanic glasses of this age is exceptionally challenging due to glass annealing under ambient conditions. The Giraffe maar chronology was revised by Buryak *et al.* (2024), who used tephra zircon U–Pb dating to show that the maar sediment fill was instead deposited ~48 Ma; a lower tephra near the base of the lacustrine silts was dated to 48.72 ± 0.30 Ma and tephra near the lacustrine–peat transition were dated to 47.995 ± 0.082|0.087 Ma. These radiometric ages suggest that the lacustrine sediments began to accumulate soon after emplacement of the kimberlite and continued over ~700 ky, followed by a period of peat deposition as the crater basin filled.

## MATERIALS AND METHODS

Mudstone chips (0.5–1.0 g) from sediments throughout the Giraffe pipe core (Fig. 2A) were oxidized using 30 % H_2_O_2_ under low heat (~70 °C) for a minimum of an hour, rinsed multiple times with distilled water, and the resulting slurries stored at 4 °C in glass vials. This oxidation procedure was sufficient to separate microfossils from the rock matrix for most samples. Some samples were heated for an additional hour, or additionally oxidized with a sulfuric acid–potassium dichromate solution (Marsicano and Siver, 1993) for further dissociation of the microfossils from the rock matrix. Aliquots of each slurry were diluted with distilled water and air-dried onto a piece of heavy-duty aluminium foil. These aluminium foil samples were trimmed, attached to aluminium scanning electron microscope (SEM) stubs with Apiezon^®^ wax, coated with a mixture of gold and palladium for 2 min with a Polaron Model E sputter coater, and observed with a FEI Nova field emission SEM. Separate aliquots of diluted slurry were air-dried onto circular glass coverslips, which were mounted onto glass slides using Naphrax and examined with a Leica DMR light microscope equipped with a Zeiss Axiocam 506 colour camera. Measurements of phytolith diameter (*n* = 30), tubercle base diameter (*n* = 15) and tubercle height (*n* = 15) were made with SEM images.

Organic remains from multiple samples containing individual phytoliths were further examined for the presence of stegmata. These samples were prepared as above, but under lower heat and approximately half of the time. In addition, portions of leaves from *Trachycarpus fortunei* were used to image both modern phytoliths and their arrangement in stegmata. Pieces of leaves were oxidized with a strong sulfuric acid–potassium dichromate solution to remove all organic components. The resulting slurry was washed with distilled water and prepared for observation of siliceous phytoliths with SEM as noted above. To observe *in situ* stegmata, small pieces of the leaves were mounted onto aluminium stubs using Apiezon^®^ wax with the undersurface facing upwards, and observed with SEM under low vacuum.

## RESULTS

### Description of fossil palm phytoliths

**Fig. 3.**
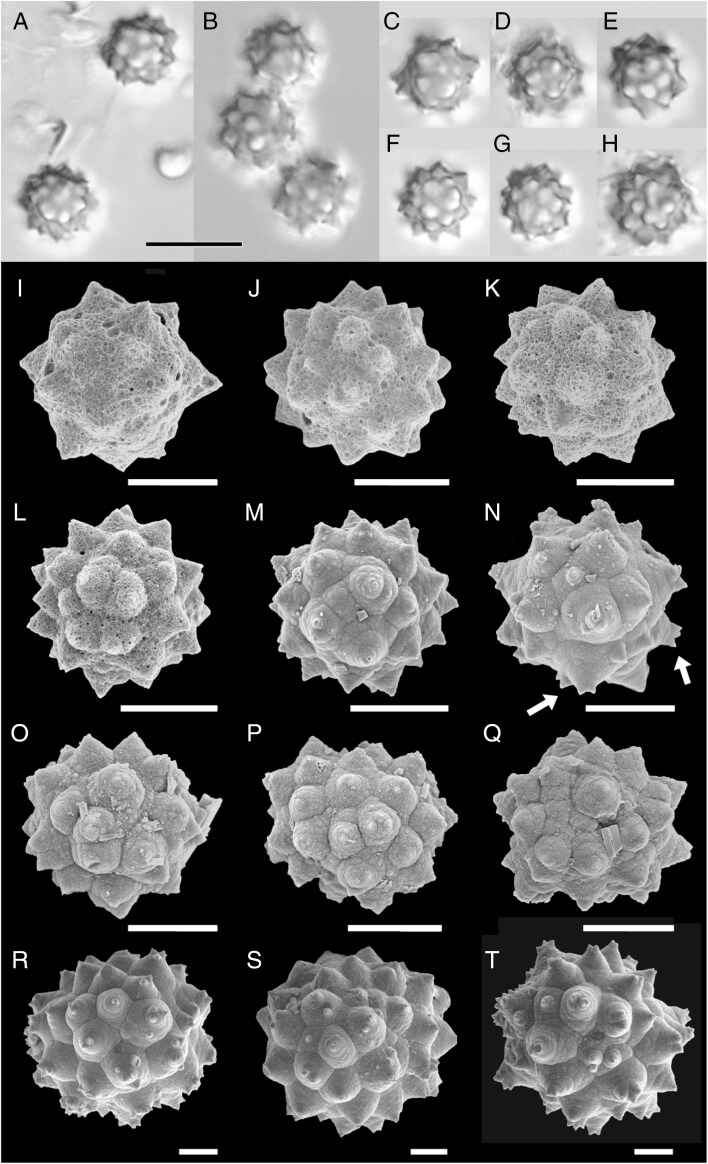
Palm phytoliths from the 48 Ma Eocene Giraffe locality. (A–H) Light microscopy (×1000 under oil immersion) of typical palm phytoliths from the Giraffe lacustrine sequence showing consistent globular echinate morphology. (I–Q) Scanning electron microscopy of Giraffe palm phytoliths showing the range of preservation and degrees of symmetry with respect to peripheral projections. White arrows in N indicate tubercles with multiple apical points. (R–T) Modern phytoliths extracted from foliage of the coryphoid palm *Trachycarpus fortunei*. Scale bars are 10 µm for light microscopy, 3 µm (I–Q) and 2 µm (R–T).

The fossil palm phytoliths are of the spheroid echinate type, radially symmetrical, and with more or less evenly spaced peripheral projections, or tubercles (Fig. 3A–Q). Practically all specimens have a spheroid bauplan, although a few are slightly elongate (Fig. 3P, Q), and the overall morphology is clearly visible in both light microscopy and SEM. Except for one specimen that was 8 µm, phytoliths range in diameter from 4 to 7.3 µm, with a mean of 5.7 µm. The tubercle projections are cone-shaped with a wide and rounded base, and taper to a sharp point. The tubercle bases are abutted over the entire phytolith surface (Fig. 3I–Q). The mean diameter of the base and height of tubercles were 1.3 and 1.1 µm, respectively. Most tubercle projections terminate in a single point; however, a second or even third point forms on some specimens as a result of additional points of silica deposition developing at the tip (e.g. Fig. 3N, arrows). The phytoliths exhibit a range of preservation states. Approximately half of the specimens show some silica dissolution (e.g. Fig. 3I–L), while the remaining half are well preserved with little to no silica erosion (e.g. Fig. 3M–Q). Preservation states could only be discerned with SEM.

### Formation of stegmata

**Fig. 4.**
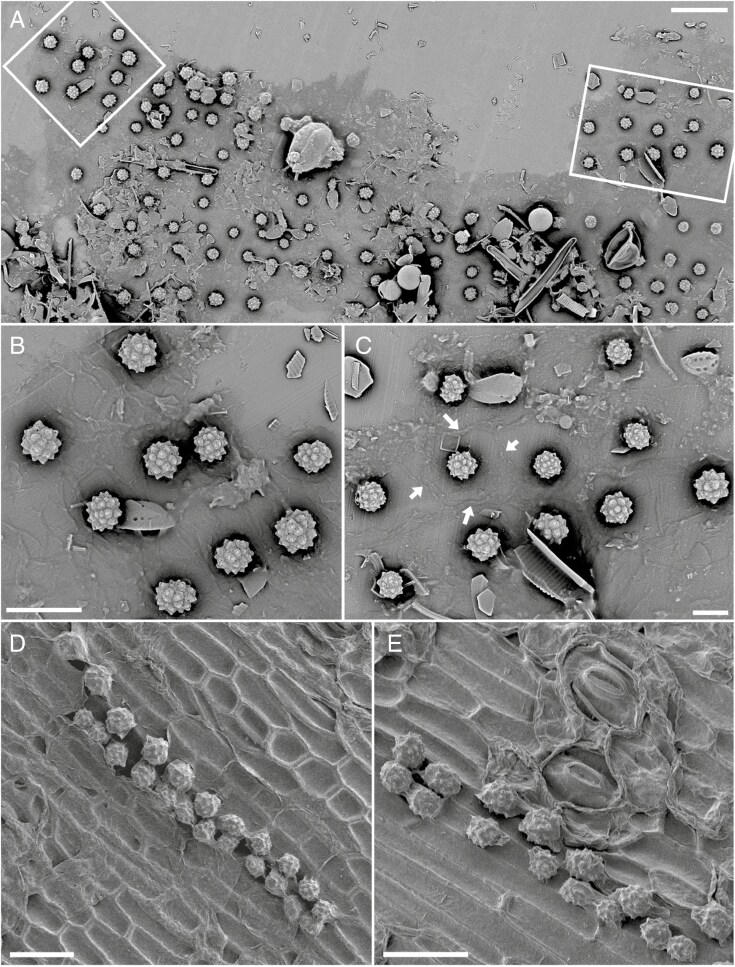
Phytolith stegmata. (A) Large remnant of phytolith stegmata (dark area) from the Giraffe material, with sub-linear files of phytoliths attached. White boxes in the upper left and right are enlarged in (B) and (C), respectively. (D, E) Stegmatal vestiges in modern leaf tissue of *Trachycarpus fortunei*, adpressed to epithelial and stomatal cells. Arrows in (C) indicate residual cell margins around one phytolith. Scale bars are 20 µm (A, D, E), 10 µm (B) and 5 µm (C).

Several mudstone samples containing dark brown mudstone with high organic content also harboured phytolith remains. These strata contained not only isolated phytoliths, but also remains of linear arrays of specimens (Fig. 4A–C). Close inspection revealed remains of cell walls faintly outlining individual cells, with each containing a phytolith, and collectively forming stegmata (e.g. Fig. 4C, arrows). One especially large ‘organic skin’, noted by the dark outline of material in Fig. 4A, contained numerous phytoliths, many of which were still aligned in stegmata.

### *Comparison with phytoliths in foliage from* Trachycarpus fortunei

Phytoliths were extracted from the foliage of a hardy outdoor specimen of *T*. *fortunei*, (Royal Botanic Garden, Edinburgh) and pieces were also observed directly to study stegmata. Phytoliths were similar in all respects (size, shape and tubercle structure) to fossil specimens from the Giraffe locality. The phytoliths had a similar spheroid echinate morphology with cone-shaped tubercles radiating from the surface (Fig. 3R–T). The tubercles also had a wide rounded base and one or two apical points. Pairs of stegmata were evident in epidermal cells on the undersurface of the leaf (Fig. 4D, E). The phytoliths within stegmata were closely spaced with one or more specimens per cell.

### Distribution of palm phytoliths and other warm-water organisms

In addition to palm phytoliths, the Eocene sediments at the Giraffe locality also contain remains of four other organisms representing aquatic organisms that today are largely restricted to tropical and subtropical environments (Fig. 2B). These included extensive specimens of the tropical synurophyte *Mallomonas bangladeshica* (Siver and Wolfe, 2009), multiple species of the warm-water diatom genus *Actinella* (Siver *et al.*, 2010, 2015), and a member of the tropical sponge family Potamolepidae, *Potamophloios canadensis* (Pisera *et al*., 2013). In addition, remains of a lesser number of specimens of another second warm-water synurophyte, *Mallomonas multiunca* v. *pocosinensis*, were recorded from a few scattered strata.

Palm phytoliths were recorded in mudstones from 45 strata spanning 37 m of the core (Fig. 2B). Three of the warm-water organisms were also abundant and widely distributed in different parts of the lake history. *Potamophloios canadensis* remains were found in 18 samples largely restricted to the early phases of the lacustrine ecosystem, including in deeper sections of the core than the part containing the palm remains. *Mallomonas bangladeshica* was an important component of the lake close to its inception, and again towards the end of its existence, reported in 41 strata. Multiple species of *Actinella* were common in 23 strata, mostly from middle stages of the lake phase. Collectively, the warm-water aquatic taxa spanned the entire time period represented by the palm remains (Fig. 2B).

## DISCUSSION

Reconstructions of the global mean annual surface temperatures during the Eocene epoch (56–34 Ma) indicate warm conditions, significantly higher than experienced today (e.g. Zachos *et al*., 2001; Sluijs *et al*., 2009; Westerhold *et al*., 2020; Judd *et al*., 2024). Temperature peaks occurred at the Palaeocene–Eocene Thermal Maximum (PETM, ~56 Ma), and the early Eocene Climatic Optimum (~53–47 Myr) (e.g. Westerhold *et al*., 2020). Despite an additional and relatively short-lived peak at the Middle Eocene Climatic Optimum (~40 Ma; Bijl *et al*., 2010), the mean global surface temperature began to decline after the Early Eocene Climatic Optimum, with a precipitous decline at the Oligocene–Eocene boundary (~34 Ma) (Zachos *et al*., 2001, 2008). While there is greater agreement that the early Eocene was an ice-free greenhouse world (e.g. Sluijs *et al*., 2009), the state of the cryosphere during the middle and late Eocene remains contested (e.g. Tripati *et al*., 2005; Stickley *et al.*, 2009; DeConto *et al*., 2012; Carter *et al*., 2017; Tripati and Darby, 2018; Stein, 2019), leaving uncertain whether or not glaciers, sea ice, permafrost, or all three were dynamic components of the climate system during this transitional climate state.

The extent of winter freezing conditions and ultimate formation of perennial ice over geological time can be aided by tracking the locations and timing of fossil Arecaceae remains. Today, there are no palm species naturally occurring in regions where the CMMT is below 5 °C (Greenwood and Wing, 1995). In addition, elevated CO_2_ concentrations are known to increase the freezing sensitivity of plants (Royer *et al*., 2002), meaning the presence of palms would likely indicate CMMT estimates above 5 °C. Remains of fossil palms have been found at multiple Cretaceous sites in North America spanning a time period ranging from ~89 Ma to the early Palaeocene (~65 Ma), inferring a warm climate with mean winter air temperature above freezing (Greenwood and Wing, 1995; Greenwood and West, 2017; Reichgelt *et al*., 2018; Greenwood *et al.*, 2022). Knowing to what extent palms inhabited landscapes in younger time periods (e.g. the Eocene), especially at more northern latitudes, would further our understanding of the timing and extent of freezing conditions, and help to assess climate models (e.g. Zhu *et al.*, 2024).

Additional macroflora records of palms have been recovered from western North America that range from the end of the Cretaceous into the Eocene, and extend the presence of the Arecaceae to ~55° N latitude with some uncertainty regarding palaeolatitude for sites in Alaska and British Columbia (Fig. 1A; Wolfe, 1977; Schweitzer, 1980; McIver, 2002; Sunderlin *et al.*, 2014; Allen, 2015; Greenwood and West, 2017; Greenwood *et al*., 2022). In addition, examining drill cores taken from the Lomonosov Ridge in the Arctic Ocean, Sluijs *et al.* (2009) reported palm pollen in the strata that represented the Eocene Thermal Maximum 2 event at 53.5 Ma. Based on their findings, they estimated that the sea surface temperature of the Arctic Ocean had increased by 3–5 °C during this episodic warming event. They further reasoned that the pollen was derived from surrounding land masses, and the presence of palms would demand the CMMT be >5 °C. In fact, they further argued that the CMMT was likely closer to 8 °C given high concentrations of CO_2_. Putative palm pollen was also reported from early Eocene strata on Ellesmere Island, High Arctic Canada (Harrington *et al.*, 2012).

In addition to the early Eocene Lomonosov Ridge and Ellesmere Island palm pollen records above 75°N, ‘palm/cycad’ pollen has also been uncovered from ~75°N latitude in early and middle Eocene sediments at ODP Site 913, Norwegian–Greenland Sea (Eldrett *et al*., 2009). Both of these plant groups indicate CMMT >5 °C. The reconstructed flora was described as one typical of lowland wetland swamps found in the south-eastern USA. However, in younger sediments from ODP sites 643A and 985A further south in the Norwegian–Greenland Sea, close to the Eocene–Oligocene boundary and estimated as being 38–30 Ma, the palm/cycad pollen had disappeared, in agreement with the cooling conditions occurring by this point in geological time (e.g. Zachos *et al*., 2001) and presence of winter freezing conditions.

Our record of palm phytoliths from the Eocene Giraffe site further extends the distribution of palms and inferred temperate climates in central/western North America to subarctic latitudes ~65° N, and at a well-constrained younger age of 48 Ma. Indeed, the mean inferred MAT, CMMT and warm month mean temperature (WMMT) values of 14.5 ± 1.3, 3.4 ± 1.7 and 24.5 ± 0.8 °C, respectively (Wolfe *et al*., 2017), confirm the assignment of a warm subtropical climate for the Giraffe locality at ~48 Ma. In addition to being significantly warmer, the inferred MAP for the Giraffe locality ranged from 1257 to 1292 mm with a mean uncertainty of ~300 mm, 4 times higher than the present. It should be noted that the palaeoclimate estimates reported by Wolfe *et al*. (2017) were based on terrestrial material in peat that was younger than the deeper lacustrine mudstones that contain the palm phytoliths. The viability of palms over a large section of the older lacustrine portion of the core supports the idea that the CMMT during this time period was slightly higher than that inferred from the overlying and younger terrestrial sediments. Indeed, Martínez-Sosa *et al*. (2023) analysed glycerol dialkyl glycerol tetraethers (GDGTs) in Giraffe maar lacustrine and peat sediment, and inferred slightly higher MAT from lacustrine sections of the core.

Freshwater sponges, synurophyte algae and diatoms whose modern counterparts are found largely in tropical and subtropical localities are also well represented throughout the Giraffe lacustrine sediments, supporting the warm, ice-free climate inferred by palm phytolith remains. The sponge family Potamolepidae, represented at the Giraffe site by *Potamophloios canadensis* (Pisera *et al*., 2013), is almost exclusively distributed in the tropics, found widespread throughout Neotropical and Afrotropical regions (Copeland *et al*., 2015). None of the localities harbouring potamolepid sponges, including the only known record outside of the tropics (Copeland *et al*., 2015), experience winter freezing. Remains of the synurophyte alga *Mallomonas bangladeshica*, a warm-water taxon classified as endemic to tropical regions (Cronberg, 1989; Kristiansen, 2002), were documented in numerous sections throughout the lacustrine sequence (Siver and Wolfe, 2009). Populations of *Mallomonas multiunca* var. *pocosinensis*, a rare synurophyte known only from ponds along coastal North Carolina that lack winter ice (Siver, 2003), were also recorded in Giraffe sediments. Lastly, five new species of the diatom genus *Actinella*, all with simple head pole morphologies, have been described from the Giraffe lacustrine sediments (Siver *et al*., 2010, 2015). A review of 58 recognized living species of *Actinella* confirms that 54 are distributed in tropical and subtropical regions of South America, Africa and Australia (Kociolek *et al*., 2001; Sabbe *et al*., 2001; Siver and Wolfe, 2009). None of the living species with simple head pole morphology, similar to those described from Giraffe, are found outside of tropical and subtropical regions. The totality of palms, coupled with the presence of tropical to subtropical freshwater organisms, not only confirms a warm climate lacking winter ice at the Giraffe locality, but implies that tropical freshwater and terrestrial organisms can persist in subarctic regions under substantially warmer-than-present climate conditions.

Though phytoliths have been effectively used in palaeo-ecological reconstructions especially for tracing, for example, development of grasslands (Strömberg, 2004; Strömberg and McInerney, 2011) and examination of Holocene vegetation patterns (Liu *et al*., 2023), the ~48 Ma Eocene Giraffe locality represents one of the oldest records of palm phytoliths. In addition, the remains of phytoliths in stegmata illustrating their original positions in foliage cells is the first such report of these structures from the fossil record. The fact that remains of the cell walls can still be observed surrounding individual phytoliths further illustrates the impressive preservation of fossils at the Giraffe site. It further suggests that ancient lacustrine remains that harbour other siliceous microfossils, such as chrysophytes and diatoms, may yield prime sites to also search for phytoliths.

Palm phytoliths have a potential advantage over pollen in palaeo-ecological reconstructions. Records of palms in oceanic cores, especially those based on small (trace) percentages of pollen, need to be viewed with caution since pollen can be transported hundreds of kilometres by wind and water currents (Hjelmroos, 1991; Robledo-Arnuncio, 2011), rendering the precise source locality unknown and potentially far removed from the core site. In contrast, the presence of palm phytoliths infers a local source, in this case close to the Giraffe maar crater.

Remains of palms have been reported from many fossil sites, and it is well known that the Arecaceae was distributed worldwide by the Paleogene (Greenwood and West, 2017; Brightly *et al*., 2024). Although the majority of palm fossils are pollen, stem or leaf remains, it is difficult to use these plant parts to identify lower taxonomic units, which in turn reduces their use in reconstructing past environments (Witteveen *et al*., 2022; Brightly *et al*., 2024). As a result, recent efforts have attempted to use phytoliths to narrow identifications to the subfamily, tribe and even genus levels. Willeveen *et al*. (2022) produced a key using a few morphological traits, but it was based on a limited number of palm species from a specific geographical region. Although Brightly *et al*. (2024) incorporated many more species and morphometric traits, and linked their findings to a well-resolved molecular phylogeny, they reported weak correlations with tribe-level units. Accordingly, we hesitate to provide confident tribe-level identification solely from phytolith traits, especially given the age of our Eocene specimens. Based on Brightly *et al*. (2024), phytoliths from Giraffe are similar to ones found within the Calamoideae, Areceae and Trachycarpeae clades. By using additional environmental data, the argument is made that Giraffe phytoliths were produced by species within the Trachycarpeae clade. Brightly *et al*. (2024) reported that most ancestral members of the Calamoideae and Areceae clades evolved under relatively warm winter conditions, significantly above freezing. In contrast, the ancestral minimum coldest month temperatures for many genera in the Trachycarpeae were ~8 °C, and several near 4 °C, much closer to the CMMT inferred for the Giraffe locality using palynology (Wolfe *et al*., 2017).

In summary, the presence of palm phytoliths over an extensive portion of the long core taken at the Giraffe kimberlite maar locality supports a warm temperate climate with mean winter temperatures above freezing at this interior continental site situated close to the Arctic Circle in the Eocene at ~48 Ma. These inferences are supported by the presence of additional fossil remains representing multiple aquatic organisms typically distributed in warm tropical and subtropical regions. The fossil phytoliths were similar in morphology to those produced by modern palms belonging to the subfamily Coryphoideae, and in particular the Trachycarpeae tribe. Lastly, recovery of stegmata demonstrated the exquisite preservation of fossils at the Giraffe locality, and further documents the evolution of such structures in Arecaceae by the late early Eocene.
